# Functional annotation of the T‐cell immunoglobulin mucin family in birds

**DOI:** 10.1111/imm.12607

**Published:** 2016-06-20

**Authors:** Tuanjun Hu, Zhiguang Wu, Lonneke Vervelde, Lisa Rothwell, David A. Hume, Pete Kaiser

**Affiliations:** ^1^ The Roslin Institute and Royal (Dick) School of Veterinary StudiesUniversity of EdinburghMidlothianUK

**Keywords:** chicken, comparative immunology, TIM1, TIM4

## Abstract

T‐cell immunoglobulin and mucin (TIM) family molecules are cell membrane proteins, preferentially expressed on various immune cells and implicated in recognition and clearance of apoptotic cells. Little is known of their function outside human and mouse, and nothing outside mammals. We identified only two TIM genes (*chTIM*) in the chicken genome, putative orthologues of mammalian *TIM1* and *TIM4*, and cloned the respective cDNAs. Like mammalian *TIM1*,* chTIM1* expression was restricted to lymphoid tissues and immune cells. The gene *chTIM4* encodes at least five splice variants with distinct expression profiles that also varied between strains of chicken. Expression of *chTIM4* was detected in myeloid antigen‐presenting cells, and in *γδ* T cells, whereas mammalian *TIM4* is not expressed in T cells. Like the mammalian proteins, chTIM1 and chTIM4 fusion proteins bind to phosphatidylserine, and are thereby implicated in recognition of apoptotic cells. The chTIM4–immunoglobulin fusion protein also had co‐stimulatory activity on chicken T cells, suggesting a function in antigen presentation.

**Abbreviations**
aa: amino acids
BM‐APC: bone marrow‐derived antigen‐presenting cells
BM‐Mф: bone marrow‐derive macrophage
chTIM: chicken TIM
ConA: concanavalin A
Ct: cycle threshold value
DMEM: Dulbecco's modified Eagle's medium
EST: expressed sequence tag
FCS: fetal calf serum
GM‐CSF: granulocyte–macrophage colony‐stimulating factor
Ig domain: mucin domain
IgFc: immunoglobulin Fc domain
IgV: immunoglobulin variable domain
IL‐4: interleukin‐4
mAb: monoclonal antibody
nt: nucleotides
PC: l‐*α*‐phosphatidylcholine
PE: 3‐sn‐phosphatidylethanolamine
PI: l‐*α*‐phosphatidylinositol
PS: 3‐sn‐phosphatidyl‐l‐serine
SPF: specific pathogen‐free
Th2: T helper type 2
TIM: T cell/transmembrane, immunoglobulin and mucin

## Introduction

The T‐cell/transmembrane, immunoglobulin and mucin (TIM) family are cell surface receptors that recognize phosphatidylserine but also regulate both innate and adaptive immune responses.^1^ In both mouse and human, the TIM family has multiple members (eight in mouse and three in human). Research has focused on the family members that are conserved between human and mouse – TIM1–4 in mouse (TIM5–8 are to date predicted genes only) and the three human TIM molecules (TIM1, TIM3 and TIM4). TIM2 is absent in human, although human TIM1 shares 32–42% amino acid identity with mouse TIM1 and TIM2 respectively and may fulfil the functions of both.

In mice, TIM1 was preferentially expressed on T helper type 2 (Th2) cells and reciprocally regulates Th2 responses, which have been demonstrated by different groups using agonistic/antagonistic anti‐TIM1 monoclonal antibodies (mAbs).^2^, ^3^, ^4^ TIM3 was preferentially expressed on Th1 cells; once engaged, it negatively regulated Th1 immunity.^5^, ^6^ TIM3 was also expressed on dendritic cells, where it mediated phagocytosis of apoptotic cells and contributed to antigen cross‐presentation.^7^ TIM4 was also suggested to be expressed exclusively on antigen‐presenting cells (APC), where it contributed to phagocytosis of apoptotic cells and maintenance of tolerance.^8^, ^9^ TIM4 also bilaterally regulates T‐cell responses by promoting activated T‐cell survival and expansion^10^, ^11^ but suppressing naive T‐cell proliferation.^12^

The chicken is the non‐mammalian species in which the immune response is best characterized. The chicken response to pathogen challenge is broadly similar to that of mammals, in that it is capable of mounting innate immune responses that drive both inflammatory (cell‐mediated) and humoral immune responses and resultant memory. Chickens share with mammals the Th1/Th2 polarization of T‐cell cytokine profiles. The response to intracellular pathogens is dominated by interferon‐*γ* and that to extracellular pathogens by interleukin‐4 (IL‐4) and IL‐13.^13^, ^14^, ^15^ This is compelling evidence for the polarization of type 1 and type 2 adaptive immune responses extending beyond mammalian species to at least galliform birds. It remains to be determined whether this paradigm holds at the cellular and molecular levels and whether avian T helper cells can become terminally polarized to a Th1 or Th2 phenotype.

Despite the similarities, the immune organs, cells and molecules used by the chicken to mount innate and adaptive responses can differ from those in mammals. Birds lack lymph nodes, and macrophages (expressing the CSF1 receptor) appear to take the role of antigen‐trapping cells within B‐cell areas, the role of non‐haematopoietic follicular dendritic cells in mammals.^16^ Only recently has the existence of a classical Flt3‐positive ‘dendritic cell’ been inferred in the chicken,^17^ but the relative importance in immune responses is not clear. In particular, chicken Th2‐driven responses seem to be different to those of mammals. Chickens lack IgE and subclasses of IgY (the avian homologue of IgG); functional eosinophils appear to be absent; the eotaxins and the eotaxin receptor are absent;^18^ IL‐5 mRNA expression is switched off during Th2 responses^15^ and Th2‐associated allergies have not been described for birds.

As Th1/Th2 polarization is apparently shared to a degree between birds and mammals, as is the clearance of dying cells by phagocytes,^16^ we aimed to identify the repertoire and biological function of the TIM family of molecules in the chicken.

## Materials and methods

#### Chicken tissues and cells

Chicken line 7_2_ was bred and maintained under specific pathogen‐free (SPF) conditions at the Institute for Animal Health. J line was intercross‐bred from nine lines, originally inbred from Brown Leghorn chickens at the Poultry Research Centre, Edinburgh, and conventionally raised at The Roslin Institute. Line7_2_ was bred by trait of resistance to pathogens^19^ and J line to study a variety of traits, such as egg laying, plumage and vigour (http://www.narf.ac.uk/chickens/lines). These two lines were chosen for this study because of their clear genetic background and diversity of breeding and in the hope of finding out whether genetic diversity has any effect on chicken TIM family molecules. Tissues were removed from 6‐week‐old chickens, either line 7_2_, or J line, without or with standard vaccine immunizations respectively, specifically thymus, spleen, bursa of Fabricius, Harderian gland, caecal tonsil, Meckel's diverticulum, bone marrow, brain, muscle, heart, liver, kidney, lung, skin, small intestine and testis. Lymphocyte subsets (CD3^+^, CD4^+^, CD8*α* ^+^, CD8*β* ^+^, TCRα*β* ^+^, TCRγδ^+^, Bu‐1^+^) and KUL01^+^ myeloid cells were isolated from total splenocytes pooled from three birds, as previously described.^20^ Bone marrow‐derived APC [BM‐APC, grown in granulocyte–macrophage colony‐stimulating factor (GM‐CSF)/IL‐4] and macrophages (BM‐MФ, grown in CSF1) were isolated from two femurs from one bird and peripheral blood monocyte‐derived macrophages were isolated from a pooled blood sample from three birds of 4‐ to 8‐week‐old line 7_2_ or J line chickens; as described previously,^20^, ^21^, ^22^ and stimulated with lipopolysaccharide (Sigma‐Aldrich, Dorset, UK) or recombinant chicken CD40 ligand (rchCD40L) for 24 hr. BM‐APC are preferred to bone marrow‐derived dendritic cells, as used previously, because it is unclear whether classical Flt3‐dependent dendritic cells are present in birds and despite their APC activity, the phenotype of the BM‐APC is primarily macrophage‐like.

#### Cloning and sequencing of cDNA

Total RNA (5 μg), extracted from the spleen of a 6‐week‐old line 7_2_ bird, was reverse‐transcribed into cDNA using a Superscript II kit (Invitrogen, Paisley, UK) to use as a template to amplify the chTIM1 cDNA by PCR; sequence‐specific primers TIM1‐F1/R1 (see Table 1) and 0·625 U Go*Taq* ^®^ DNA polymerase (Promega, Southampton, UK) were used in a 20‐μl total volume with the PCR as follows: after denaturation of the cDNA template at 94° for 3 min, a touch‐up PCR was carried out with cycling conditions of 94° for 1 min, 50° for 30 seconds and 72° for 90 seconds for three cycles; 94° for 1 min, 52° for 30 seconds and 72° for 90 seconds for three cycles; 94° for 1 min, 54° for 30 seconds and 72° for 90 seconds for 30 cycles. The first‐round PCR product was then used as template for amplification with a nested primer pair, TIM1‐F2/R2. Cycling conditions were 94° for 3 min, followed by 34 cycles of 94° for 1 min, 50° for 30 seconds and 72° for 2 min.

**Table 1:** Primers use to amplify chicken T‐cell immunoglobulin and mucin (chTIM) family cDNAs and to generate expression constructs

Primer	Sequence (5’–3’)
TIM1‐F1	AATAAGAGTGTTCCTCATC
TIM1‐R1	AAGTTTTTAATTAGAATTGTAGC
TIM1‐F2	ATGTCTTCTCATTTCTTCC
TIM1‐R2	TTAATTAGAATTGTAGCTTTTATTTG
TIM4‐F1	AGCCAAAATGTCCCACTTT
TIM4‐R1	ACACAGATCCCAGAAATACTACTG
TIM4‐F2	ATGTCCCACTTTGTGTTGTTTC
TIM4‐R2	TCACAGCACAAAAAGGTTGT
TIM1‐IgVF	***GCTAGC***ATGTCTTCTCATTTCTTC
TIM1‐IgVR	***AGATCT***TCAACCACCACCTGGAGGTT
TIM1‐mucinF	***GCTAGC***AGAGCTAGGGTCTCTACT
TIM1‐mucinR	***AGATCT***TTTTCTGAATACTGCTGACT
TIM4S‐IgVF	***GCTAGC***ATGTCCCACTTTGTGTT
TIM4S‐IgVR	***AGATCT***AGCACCACCAGCTGAATGTT
TIM4S‐mucinF	***GCTAGC***GAAGCACCTCCATTGATG
TIM4S‐mucinR	***AGATCT***GGAAAGGAAAGTTTCATCCC

Nucleotides in bold italics show the *Nhe*I or *Bgl*II restriction enzyme sites introduced to facilitate directional cloning into the vectors pKW06‐Ig and BR‐Ig.

For amplification of chTIM4 cDNA, bone marrow was isolated from a 6‐week‐old J line bird and cells were purified as previously described;^21^ total RNA (2 μg), extracted from the cells, was reverse‐transcribed into cDNA as before. The chTIM4 cDNA was amplified, using sequence‐specific primer pair TIM4‐F1/R1 with a touch‐down PCR with cycling conditions of 94° for 3 min; 94° for 1 min, 60° for 30 seconds and 72° for 2 min for three cycles; 94° for 1 min, 58° for 30 seconds and 72° for 2 min for three cycles; 94° for 1 min, 57° for 30 seconds and 72° for 2 min for 30 cycles. Again the first‐round PCR product was used as template for amplification with a nested primer pair, TIM4‐F2/R2. Cycling conditions were 94° for 3 min; 94° for 1 min, 60° for 30 seconds and 72° for 2·5 min for three cycles; 94° for 1 min, 61° for 30 seconds and 72° for 2·5 min for 30 cycles.

The cDNAs were initially cloned into pGEM‐T Easy (Promega) and sequence verified. The complete amplified cDNA sequences of both genes were submitted to the EMBL Nucleotide Sequence Database with accession numbers of HG425163 for chTIM1, LN831082 for chTIM4L_0_, HG425165 for chTIM4L_1_, LN831081 for chTIM4L_2_, HG425164 for chTIM4 and LN831080 for chTIM4S.

#### Real‐time quantitative RT‐PCR analysis of chTIM family member expression

RNA from the tissues and cells described above was extracted using an RNeasy Mini kit (Qiagen, Manchester, UK) following the manufacturer's instructions. TaqMan real‐time quantitative RT‐PCR was used to quantify the mRNA levels of the chTIM family members. Primers and probes specific to the different chTIM family members (Table 2) were designed using primer express (Applied Biosystems, Paisley, UK). Assays were performed using the TaqMan Fast Universal PCR master mix and one‐step RT‐PCR master mix reagents (Applied Biosystems). Data are expressed in terms of the cycle threshold value (Ct), normalized for each sample using the Ct value of 28S rRNA product for the same sample, as described previously.^14^, ^15^, ^23^

**Table 2:** Real‐time quantitative RT‐PCR probes and primers

RNA target	Probe/primer sequence (5’–3’)	Exon boundary
28S	Probe (FAM)‐AGGACCGCTACGGACCTCCACCA‐(TAMRA)	
	F GGCGAAGCCAGAGGAAACT	
	R GACGACCGATTTGCACGTC	
chTIM1	Probe (FAM)‐TGGCTTACAGGCCCCACAGTGTCG‐(TAMRA)	1/2/3
	F TCCTGGACTGGATCCTTCTGA	
	R TCTGACCAACCTCTCCCTTCA	
chTIM4 isoforms	Probe (FAM)‐ACTGTAGCAACAGCACTGCCAGAGCCA‐(TAMRA)	7/8
	F AGTTCTCTCTTGGCAGATGATGTG	
	R AAGGGTTAACTTCGGTGTCTGAAG	

F, forward primer; R, reverse primer.

#### Expression of recombinant proteins in COS‐7 cells

The different domains of chTIM1 and chTIM4 were amplified using the primer pairs shown in Table 1 and subcloned into expression vectors for expression as IgFc‐fusion proteins. For chTIM1, the IgV domain (amino acids 1–130) and the full extracellular domain (amino acids 1–207) were subcloned into the vector pKW06‐Ig.^24^ The mucin domain (amino acids 131–207) was subcloned into the vector BR‐Ig.^25^ For the chTIM4 isoforms, the IgV domains (amino acids 1–130 for chTIM4, 1–316 for chTIM4L_1_) and the full extracellular domains (amino acids 1–299 for chTIM4, 1–497 for chTIM4L_1_) were subcloned into pKW06. The mucin domain (amino acids 131–299 from chTIM4) was subcloned into BR‐Ig. All of the recombinant plasmids were sequence‐verified.

The different chTIM IgFc fusion proteins were expressed in COS‐7 cells using a well‐described DEAE‐dextran transfection method.^26^, ^27^ Successful expression was confirmed with a sandwich ELISA using unlabelled and horseradish peroxidase‐labelled goat anti‐human IgG (SouthernBiotech, Cambridge, UK) as capture and detection reagents,^24^ and the relative protein concentration estimated by comparison with a human IgFc protein standard (R&D Systems, Abingdon, UK).

The above transfection procedure was repeated several times to yield large volumes of supernatant for each recombinant TIM IgFc protein. The fusion proteins were purified on protein G columns (GE Healthcare, Amersham, UK) following the manufacturer's instructions.

#### Characterization of chTIM family molecule ligands on chicken splenocytes

To characterize the potential ligands for the chTIM family molecules, freshly isolated chicken splenocytes were incubated for 1 hr on ice with COS‐7 cell supernatants containing the recombinant IgV‐, mucin‐ and full extracellular‐domain chTIM1 or chTIM4 IgFc fusion proteins, with human IgFc protein (R&D Systems) as a control. The chTIM1 fusion proteins were adjusted to 5 μg/ml with Dulbecco's modified Eagle's medium (DMEM) plus 10% fetal calf serum (FCS), and the chTIM4 fusion proteins to 3 μg/ml. After two washes, the cells were stained with an FITC‐conjugated goat anti‐human IgG mAb (Southern Biotech) for 1 hr on ice. The staining results were analysed by flow cytometry on a FACSCalibur instrument (BD Biosciences, Oxford, UK). To determine whether activation affects expression of the chTIM4 ligands, splenocytes were co‐cultured without or with 2 μg/ml of rchCD40L or 2 μg/ml concanavalin A (ConA; Sigma‐Aldrich) for 48 hr. The cells were then double‐stained with chTIM4‐extracellular domain immunoglobulin or an immunoglobulin control (R&D Systems) fusion protein, labelled with Alexa Fluor‐647 using a Zenon labelling kit (Invitrogen), and CD3‐FITC or Bu‐1‐FITC mAbs (Southern Biotech).

#### Binding of TIM proteins to phospholipids

l‐*α*‐phosphatidylcholine (PC) from egg yolk, 3‐sn‐phosphatidylethanolamine (PE) from bovine brain, l‐*α*‐phosphatidylinositol (PI) ammonium salt solution from bovine liver and 3‐sn‐phosphatidyl‐l‐serine (PS) sodium salt from bovine brain (all from Sigma‐Aldrich) were dissolved in chloroform. The interactions between the chTIM proteins and these phospholipids were tested by a phospholipid binding assay on nitrocellulose membrane and a solid‐phase ELISA as previously described.^8^ To determine whether pre‐incubation of chTIM4 IgFc fusion proteins with PS blocks binding, splenocytes were co‐cultured without or with 2 μg/ml rchCD40L or 2 μg/ml ConA for 48 hr. PS was diluted in PBS and sonicated in an ultrasonic liquid processor (Misonix, Farmingdale, NY) at full power for 5 min to yield a 1·5 m solution. The chTIM4‐extracellular‐domain IgFc fusion protein, or recombinant chCTLA4‐extracellular‐domain IgFc fusion protein as a negative control, was diluted in DMEM plus 10% FCS, 2·5 mm CaCl_2_, with PS added at 50 nmol (0·12 μg) per μg protein and incubated for 1 hr at room temperature. The blocked and unblocked recombinant proteins were diluted to 10 μg/ml in DMEM plus 10% FCS, 2·5 mm CaCl_2_. This solution was used to immunostain the above cultured splenocytes, followed by an FITC‐conjugated rabbit anti‐human IgG polyclonal antibody (Southern Biotech), and the results were analysed by flow cytometry.

#### Co‐stimulation of splenocytes

Anti‐chicken CD3 and CD28 mAbs (Southern Biotech) were pre‐coated on a flat‐bottomed 96‐well tissue culture plate, adding 50 μl of either antibody at 1·0 μg/ml in sterile PBS, for 3 hr at 37°. The plates were then vigorously washed three times with sterile PBS. Isolated chicken splenocytes, 2·0 × 10^5^ cells/well in 100 μl medium, were cultured in this plate without or with recombinant chTIM4‐Ig protein, at different concentrations. After 48 hr of growth at 41° in 5% CO_2_, the cells were pulsed with 1 μCi of [^3^H]thymidine per well for 16 hr. Incorporation of [^3^H]thymidine (corrected counts per minute) was measured in a scintillation *β*‐counter (Perkin Elmer, Waltham, MA).

#### Monoclonal antibody generation and characterization

Fusion proteins for immunization were expressed from cloned, transfected Chinese hamster ovary cells, selected by ELISA for high‐level expression as described before^25^ and purified on protein G columns (GE Healthcare). Two Biozzi strain mice were immunized with the chTIM4L_1_‐linker‐domain (amino acids 131–207) IgFc fusion protein and three BALB/c strain mice with the chTIM4‐extracellular‐domain (amino acids 1–209) IgFc fusion protein. All mice were immunized three times with a subcutaneous injection of the immunoglobulin fusion proteins emulsified with TiterMax Gold (Invitrogen) as adjuvant. A final boost was an intraperitoneal injection of the immunoglobulin fusion proteins without adjuvant. Spleen cells were fused with Sp2/0‐Ag14 mouse myeloma cells^28^ according to standard procedures.^29^

The specificity of anti‐chTIM4 mAbs was examined by immunostaining of COS‐7 cells using anti‐chTIM4 mAbs, JH9 (anti‐chTIM4) and IE12 (anti‐chTIM4L_1_‐linker), in which the COS‐7 cells were transiently transfected with plasmids encoding full‐length cDNAs for chTIM4 and chTIM4L_1_, or empty plasmid (mock), using Lipofectine 2000 (Life Technologies, Paisley, UK) and grown for 48 hr at 37°, 5% CO_2_. The cell lysates of similarly transfected COS‐7 cells were resolved on 4–12% SDS–PAGE gels (Bio‐Rad, Hemel Hampstead, UK) for Western blot analysis and the chTIM4 proteins were probed by JH9 and IE12 mAbs.

#### Expression of chTIM4 on chicken antigen‐presenting cells

The BM‐APC, BM‐MФ and bursal cells (predominantly B cells) were immunostained with the anti‐chTIM4 mAbs (JH9 or IE12) or isotype‐matched controls. The secondary antibody was Alexa Fluor 647‐conjugated goat anti‐mouse IgG (Southern Biotech). Cells were analysed by flow cytometry using a FACSCalibur instrument (BD Biosciences) and the resulting data were analysed with flowjo software.

## Results

### The chTIM family has only two members, but chTIM4, unlike mammalian TIM4, has several isoforms

**Figure 1:**
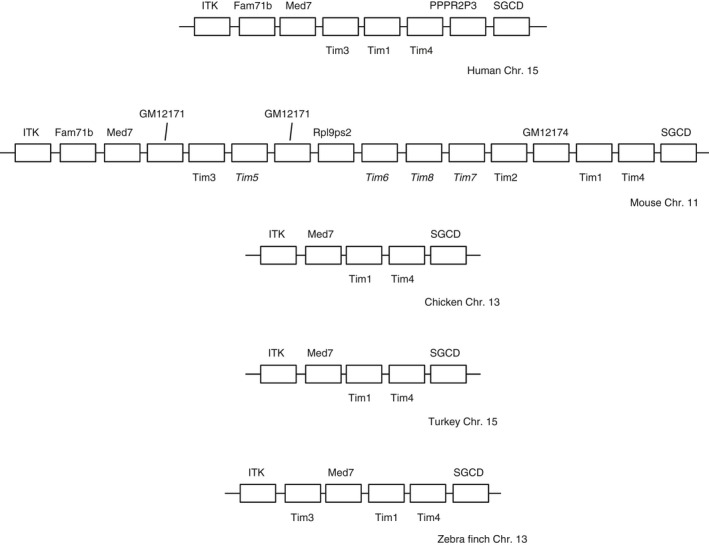
Schematic depicting the T‐cell immunoglobulin and mucin (TIM) gene family locus with a degree of conserved synteny between mammals (human and mouse) and birds (chicken, turkey and zebra finch). Human has three family members, the mouse eight, but four of these (shown in italics) are apparently pseudogenes. In contrast, the chicken and turkey both have only two members, whereas the zebra finch has three.

The amino acid sequences of human TIM1, TIM3 and TIM4 were used as probes in BLAST searches against the chicken expressed sequence tag (EST) and genome databases (v.38) in ENSEMBL. The resulting EST hits matched two clusters of overlapping chicken EST sequences, which were homologous to human TIM1 and TIM4, respectively. The chTIM family was identified on chromosome 13 by examining conserved synteny between the human and mouse TIM family clusters on chromosomes 5 and 11, respectively, and the chicken genome (Fig. 1). Only two genes were identified in the chicken. Other avian genomes were then compared; again, two genes (TIM1 and TIM4) were identified in the turkey, but three (TIM1, TIM3 and TIM4) were identified in the zebra finch (Fig. 1).

**Figure 2:**
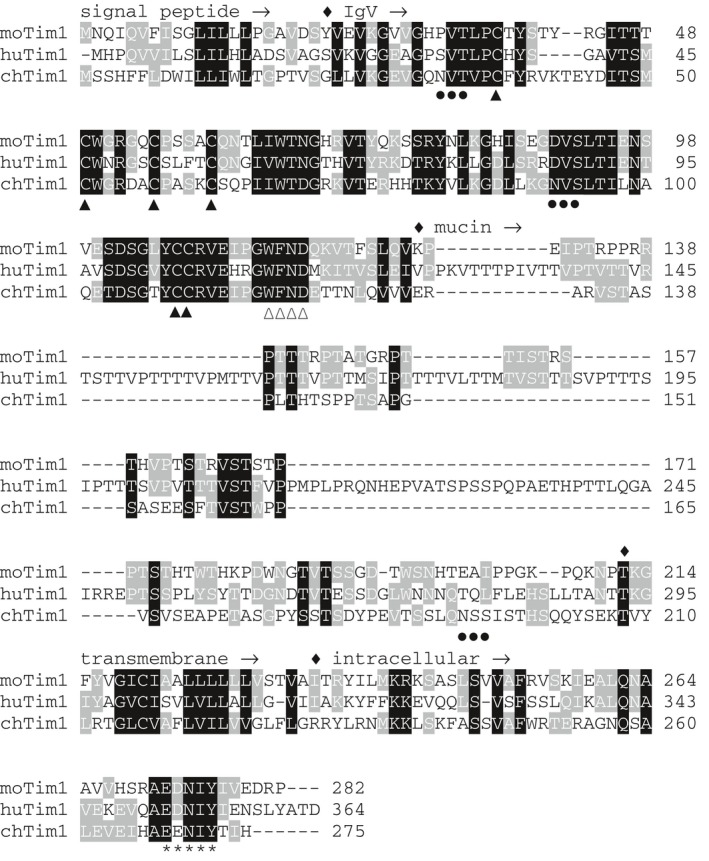
Alignment of the predicted chicken T‐cell immunoglobulin and mucin 1 (chTIM1) amino acid (aa) sequence with those of human and mouse TIM1, with reference to secondary structural features and functionally important residues. Shaded areas represent conservation of amino acid similarity – the darker the shading, the more conserved the residue across species. Dashes indicate gaps in the alignment. Filled triangles indicate the conserved six cysteines and empty triangles the conserved phosphatidylserine‐binding site (WFND). Asterisks depict the conserved tyrosine kinase phosphorylation site (AE^D^/_E_NIY). Potential N‐linked glycosylation sites in the chicken molecule are highlighted by solid dots. Filled diamonds above the sequences label the start‐points of each domain. The accession numbers of the sequences used are as follows: mouse, AAL35775; human, NP036338; chicken, HG425163.

A cDNA for TIM1 was amplified from spleen RNA. The chTIM1 cDNA consists of 828 nucleotides (nt), encoding an open reading frame of 275 amino acids (aa), with the predicted signal sequence (signalP 3.0) comprising the first 21 aa. Comparison of the predicted amino acid sequence of chTIM1 with human and mouse TIM1 (Fig. 2) revealed 32–33% amino acid identity. Overall the secondary structure of TIM1 appears conserved, with an apparent extracellular IgV domain with six conserved cysteine residues and a PS‐binding site (WFND),^9^, ^30^ a less structured extracellular mucin domain, a hydrophobic transmembrane domain and an intracellular domain containing a tyrosine kinase phosphorylation site (AE^D^/_E_NIY).^31^, ^32^

**Figure 3:**
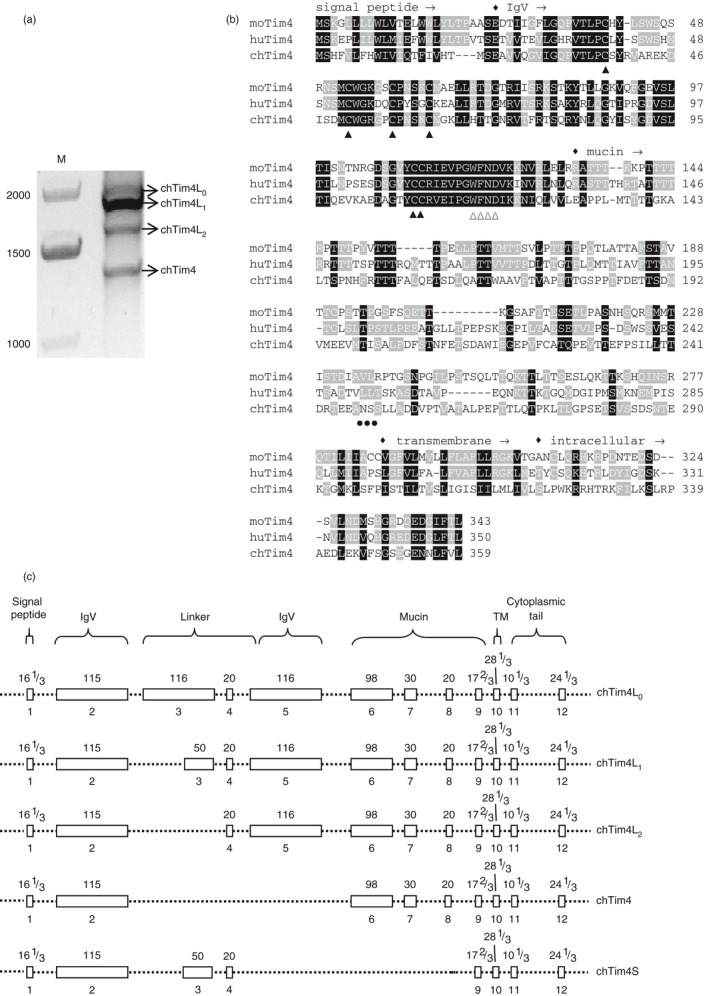
Identification and characterization of chicken T‐cell immunoglobulin and mucin 4 (chTIM4) isoforms. (a) Amplification of four isoforms of chTIM4 from bone marrow cells from a 6‐week‐old J line bird. (b) Alignment of the predicted chTIM4 amino acid (aa) sequence with those of human and mouse TIM4, with reference to secondary structural features and functionally important residues. Shaded areas represent conservation of amino acid similarity – the darker the shading, the more conserved the residue across species. Dashes indicate gaps in the alignment. Filled triangles indicate the six conserved cysteines and empty triangles the conserved phosphatidylserine binding site (WFND). Potential N‐linked glycosylation sites in the chicken molecule are highlighted by solid dots. Filled diamonds above the sequences label the start‐points of each domain. The accession numbers of the sequences used are as follows: mouse, NP848874; human, NP001140198; chicken, HG425164. (c) Structure of the chTIM4 gene showing the different exons used to encode chTIM4L_0_, chTIM4L_1_, chTIM4L_2_, chTIM4 and chTIM4S. The open reading frame (ORF) encoding chTIM4L_0_ consists of exons 1–12; exon 2 encodes an IgV domain, exon 5 a second IgV domain, and exons 3, 4 and part of exon 5 a linker between the two IgV domains. The ORF encoding chTIM4L_0_ is similar to that encoding chTIM4L_1_ but has an extended exon 3. By contrast, chTIM4L_2_ lacks the whole exon 3, chTIM4 lacks exons 3, 4 and 5, being encoded by nine exons and chTIM4S lacks exons 5–8, being encoded by eight exons. The number above each exon shows its length in aa, and that below its number.

Amplification of cDNA for TIM4 yielded multiple products (Fig. 3a). All were cloned, sequenced and shown to be isoforms homologous to mammalian TIM4; one isoform was of a similar length to mammalian TIM4, named as chTIM4. Alternative forms were either shorter [chTIM4S (short)] or longer [chTIM4L (long)]. The chTIM4L displayed additional heterogeneity, based on the length of exon 3; they were further named as chTIM4L_0_ (with the longest exon 3), chTIM4L_1_ (with a shorter exon 3) and chTIM4L_2_ (lacking exon 3).

The dominant chTIM4 amplicon consists of 1363 nt, encoding an open reading frame of 359 aa, with the predicted signal sequence (signalP 3.0) comprising the first 21 aa. Comparison of the predicted amino acid sequence of chTIM4 with human and mouse TIM4 (Fig. 3b) revealed 28–32% amino acid identity. As with TIM1, the secondary structure of chTIM4 is predicted to be similar to the mammalian proteins, with an extracellular IgV domain with six conserved cysteine residues and a conserved PS binding site (WFND),^8^, ^9^, ^30^ a less structured extracellular mucin domain, a hydrophobic transmembrane domain and an intracellular domain. The alternative chTIM4 amplicons, including chTIM4L_0_ (2099 nt), chTIM4L_1_ (1901 nt) and chTIM4L_2_ (1751 nt), generated by primer pair TIM4‐F1/R1, encoded open reading frames of 612, 546 and 496 aa respectively. Moreover, chTIM4S was amplified by a nested PCR using primer pair TIM4‐F2/R2, resulting in an 846‐nt amplicon with an encoding open reading frame of 282 aa. All the chTIM4 gene isoforms are encoded by the same gene (Fig. 3c). All chTIM4L isoforms contain two or three extra exons, encoding a second IgV domain and a linker joining it to the IgV domain that it shares with the chTIM4 isoform. The two IgV domains are very similar, differing in only 8 aa. The chTIM4L_0_ has an extended exon 3, using a later splice donor site compared with the chTIM4L_1_ isoform. Although of a similar length to exons 2 and 5, exon 3 in chTIM4L_0_ does not code a recognizable IgV domain. The chTIM4S splices out exons 5–8, but maintains the same exons 3 and 4 as those of the chTIM4L_1_ isoform.

### Tissue distribution and cellular expression of the chTIM molecules

**Figure 4:**
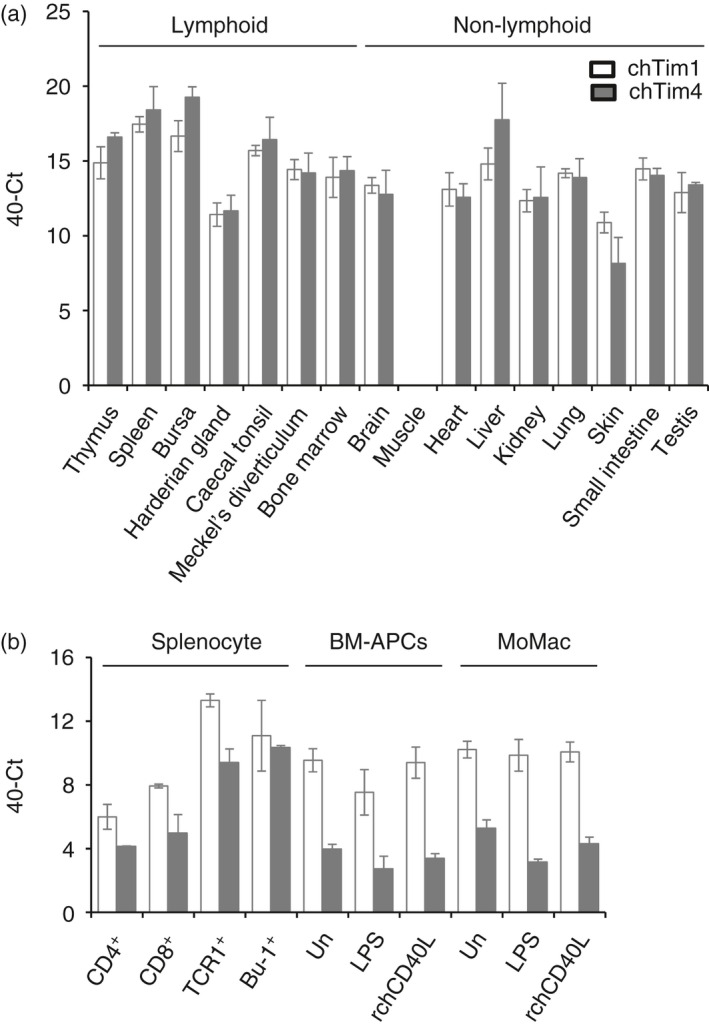
Expression patterns of mRNA of the chicken T‐cell immunoglobulin and mucin 1 (chTIM1) (blank bar) and chTIM4 (black bar), as measured by real‐time RT‐PCR, with results expressed as corrected 40‐Ct values ± SEM, in (a) lymphoid and non‐lymphoid chicken tissues from one bird, and (b) splenocyte subsets, unstimulated (Un) and stimulated bone‐marrow‐derived antigen‐presenting cells (BM‐APCs) and peripheral blood monocyte‐derived macrophages (MoMac) by lipopolysaccharide (LPS) or recombinant chicken CD40 ligand (rchCD40L) for 24 hr, in which splenocyte subsets and MoMac were prepared from a pooled sample of three birds; Assays were carried out in triplicate, and the pooled data from three experiments are shown.

Expression of chTIM1 and chTIM4 in tissues and immune cells from a line 7_2_ SPF bird was measured by quantitative PCR. By contrast to the rather restricted expression of mammalian TIM1 and TIM4 (see www.biogps.org), chTIM1 and chTIM4 were widely and similarly expressed at the mRNA level in lymphoid and non‐lymphoid tissues (Fig. 4a). The mRNAs for the chTIM1 and chTIM4 molecules were expressed in splenic CD4^+^, CD8^+^ and TCR1^+^ (*γδ*) T cells and Bu‐1^+^ B cells at variable levels. The chTIM mRNAs were also expressed in unstimulated and stimulated BM‐APC (bone marrow cells cultured in the presence of rchIL‐4 and rchCSF‐3 for 6 days) and peripheral blood monocyte‐derived macrophages. Lipopolysaccharide stimulation down‐regulated levels of mRNA expression for chTIM1 and chTIM4, whereas rchCD40L stimulation had little effect (Fig. 4b).

The cellular expression pattern of chicken TIM in J line birds was not measured. It could be different from line 7_2_ SPF birds because the rearing conditions and genetic background between them differ. On the other hand, as a receptor of TIM1, TIM4 plays crucial biological activities on TIM1‐expressing cells, including cell proliferations.^10^ The J line birds have multiple alternative splice TIM4 isoforms, therefore investigating expression pattern of its individual isoform in tissues and immune cells would be an urgent issue to be addressed.

### Divergent expression of chTIM4 isoforms in different strains of chicken

**Figure 5:**
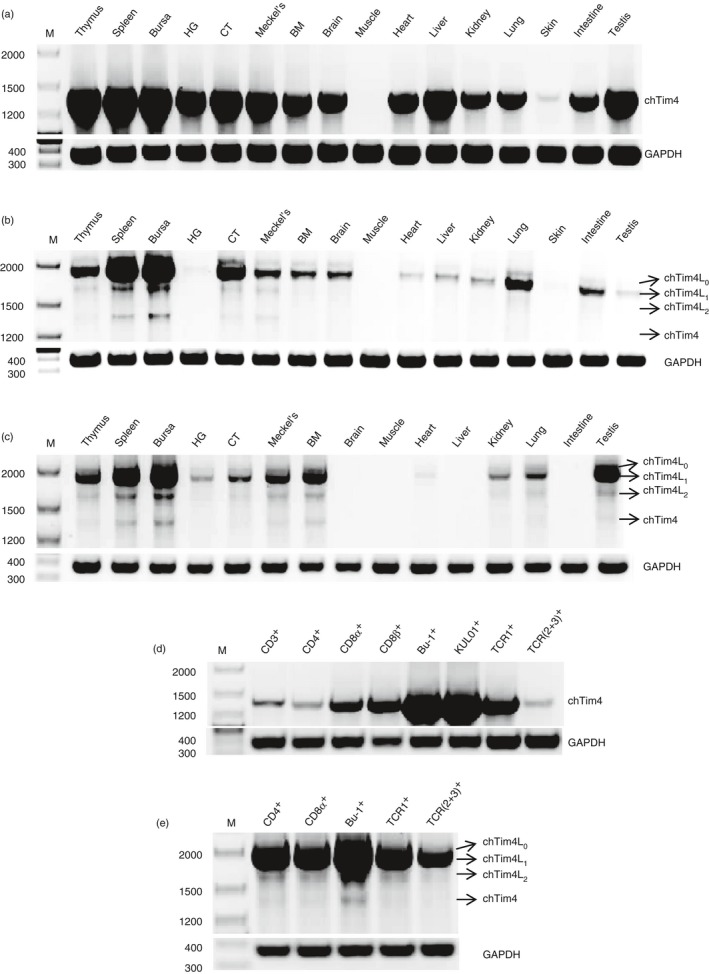
Expression patterns of the chicken T‐cell immunoglobulin and mucin 4 (chTIM4) isoforms in different strains of chicken, as measured by RT‐PCR, using primers TIM4‐F1/R1. (a) Tissues from a 6‐week‐old line 7_2_ male bird, (b) tissues from an age‐matched J line male bird with standard vaccination and (c) tissues from an age‐matched, unvaccinated J line male bird. Lymphoid and non‐lymphoid chicken tissues, where HG refers to Harderian gland, CT, caecal tonsil, BM bone marrow, intestine, small intestine. (d) Chicken splenic cell subsets from 6‐week‐old line 7_2_ birds, including: 1, CD3^+^; 2, CD4^+^; 3, CD8*α* ^+^; 4, CD8*β* ^+^; 5, Bu‐1^+^; 6, KUL01^+^; 7, TCR1^+^ (*γδ* T cells); 8, TCR(2 + 3)^+^ (*αβ*T cells). (e) Chicken splenic cell subsets from 6‐week‐old J line birds: 1, CD4^+^; 2, CD8*α* ^+^; 3, Bu‐1^+^; 4, TCR1^+^; 5, TCR(2 + 3)^+^. M represents a DNA ladder (bp) and the chicken GAPDH cDNA is a control. The results shown in (a), (b) and (c) are one representative of three birds and sorted cells in (d) and (e) were pools of three birds.

The expression patterns of chTIM4 isoforms were analysed by RT‐PCR using TIM4‐F1/R1 primers. Consistent with quantitative PCR analysis, lymphoid and non‐lymphoid tissues from a line 7_2_ SPF bird predominantly expressed the chTIM4 isoform homologous to the mammalian gene product, whereas other isoforms were undetectable (Fig. 5a). In contrast, in tissues from a conventional J line bird with a standard vaccination schedule, chTIM4L_1_ was predominantly expressed, whereas chTIM4, chTIM4L_0_ and chTIM4L_2_ were detected in some tissues, but at relatively low levels (Fig. 5b). To clarify if vaccination varies the expression pattern of chTIM4 isoforms, tissues from a non‐vaccinated J line bird were also analysed. Again, chTIM4L_1_ was the predominant form, with other chTIM4 isoforms detected at low levels (Fig. 5c). The expression of chTIM4 isoforms in different immune cells was also examined. The splenic cell subsets from line 7_2_ SPF birds consistently expressed only the chTIM4 isoform, with B cells (Bu‐1^+^) and macrophages (KUL01^+^) expressing higher levels of chTIM4 than T cells (CD3^+^) and T‐cell subsets, including CD4^+^, CD8*α* ^+^, CD8*β* ^+^, TCR1^+^ (*γδ* T cells) and TCR(2 + 3)^+^ (*αβ* T cells) (Fig. 5d). The immune cells isolated from vaccinated J line birds had the same expression pattern of chTIM4 isoforms in T‐cell subsets, including CD4^+^, CD8*α* ^+^, TCR1^+^ (*γδ* T cells) and TCR(2 + 3)^+^ (*αβ* T cells), and B cells (Bu‐1^+^) as in the J line tissue panels; i.e. chTIM4L_1_ was the predominant form (Fig. 5e).

**Figure 6:**
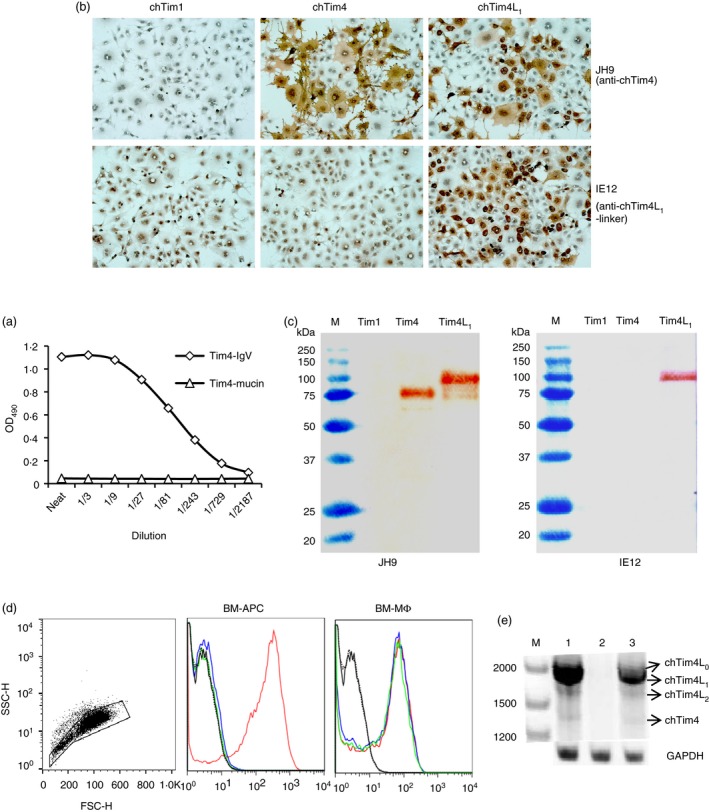
Characterization of anti‐chicken T‐cell immunoglobulin and mucin 4 (chTIM4) antibodies and chTIM4 expression in chicken antigen‐presenting cells (APCs). (a) Epitope of JH9 monoclonal antibody (mAb) was mapped to chTIM4‐IgV domain by capture ELISA, whereas chTIM4‐IgV‐Ig and chTIM4‐mucin‐Ig fusion proteins expressed by COS‐7 cells were captured by anti‐human IgFc antibody. (b) Immunostaining of COS‐7 cells with either mAb JH9 (anti‐chTIM4) or IE12 (anti‐chTIM4L1‐linker), in which the cells were transfected with plasmids expressing either full‐length chTIM4 or chTIM4L_1_ or chTIM1. AEC substrate was used to detect bound mAbs. (c) Western blot analysis of chTIM4 protein expression in transfected COS‐7 cell lysates. (d) Flow cytometrical analysis of chTIM4 expression in bone‐marrow‐derived APC (BM‐APC), bone‐marrow‐derived macrophages (BM‐Mφ) cultured from J line birds. Green, cells stained with mAb JH9; blue, cells stained with mAb IE12; red, cells stained with KUL01 (macrophages); pink, cells stained with anti‐Bu‐1 (B cells); solid black, IgG2a isotype control; dotted black, IgG1 isotype control. The cells were gated on FSC and SSC plot to exclude dead cells and cell debris. Results are representative from one of three experiments. (e) RT‐PCR analysis of chTIM4 expression in BM‐Mφ (1), BM‐APC (2) and cultured bone marrow cells only (3) as non‐treated controls. M represents a DNA ladder (bp) and the chicken GAPDH gene is a control.

To enable detection of the protein, monoclonal antibodies (mAbs) specific for chTIM4 were produced. The mAb JH9 was generated against the IgV domain of chTIM4 (Fig. 6a), and recognized all isoforms of chTIM4. The mAb IE12 was raised against the linker of chTIM4L_1_, and consequently does not cross‐react with chTIM4 (Fig. 6b,c). Neither antibody cross‐reacted with chTim1 (Fig. 6b,c). The antibodies detected a 75 000 molecular weight protein for full‐length of chTIM4 and a 100 000 molecular weight protein for full‐length of chTIM4L_1_ in transfected COS‐7 cells expressing the respective proteins (Fig. 6c). These two mAbs were used in flow cytometry to stain BM‐APC and BM‐Mφ derived from J line birds (Fig. 6d). Neither mAb stained BM‐APC, whereas both stained BM‐Mφ at equal levels. Both populations were positive for the macrophage marker, KUL01. RT‐PCR analysis confirmed that expression of chTIM4 iosforms was down‐regulated in BM‐APC, but readily detected in BM‐Mφ, when compared with untreated bone marrow cells (Fig. 6e).

### Ligands for chTIM1 and chTIM4

To study the ligands for chTIM1 and chTIM4, IgFc fusion proteins for both were expressed in transfected COS‐7 cells. Attempts to express the extracellular domain or IgV domain of chTIM4L_1_ were unsuccessful (see Supplementary material, Fig. S1). Staining of transfected COS‐7 indicated that the fusion proteins were retained in the endoplasmic reticulum (see Supplementary material, Fig. S1). We speculate that the presence of two PS binding sites in the longer protein might promote retention through binding to endoplasmic reticulum membranes.

**Figure 7:**
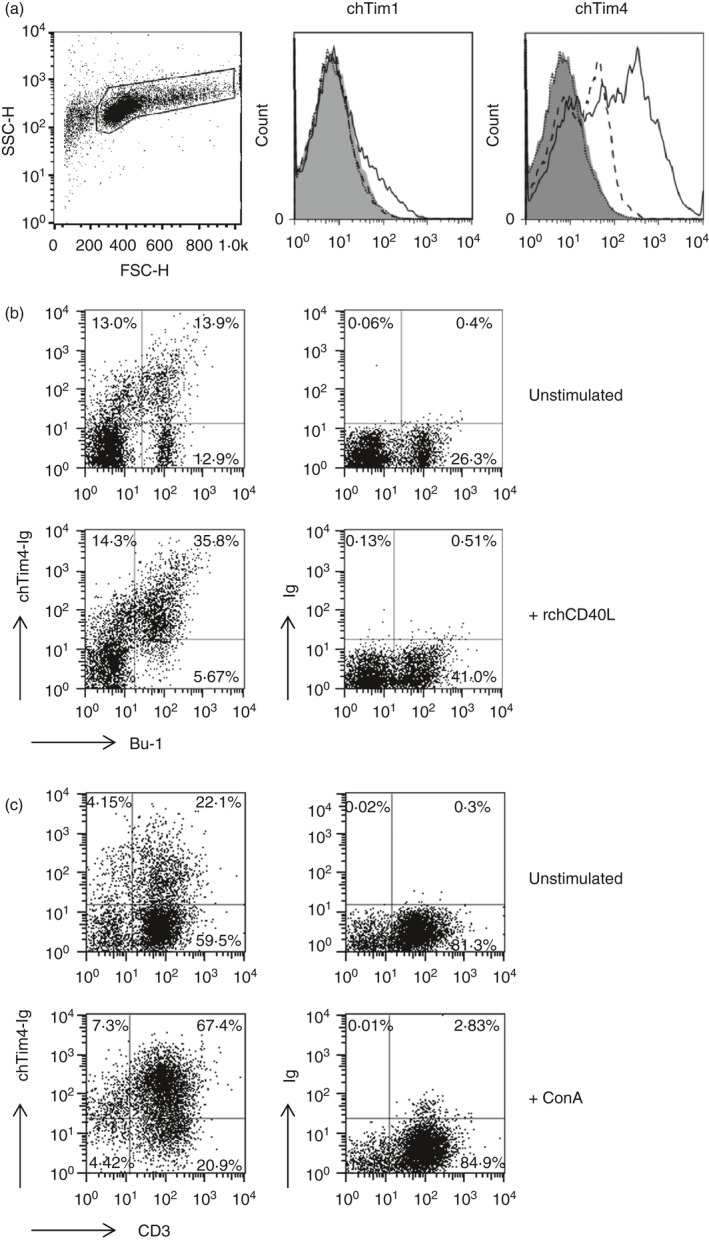
(a) The ligands for chicken T‐cell immunoglobulin and mucin 1 (chTIM1) and chTIM4 are expressed on the surface of splenocytes. Splenocytes were stained with recombinant fusion proteins of different domains of chTIM1 or chTIM4. Isolated splenocytes were stained with either human IgFc protein (grey‐filled histogram), chTIM‐IgV‐domain IgFc fusion protein (dashed line), chTIM‐mucin‐domain IgFc fusion protein (dotted line) or chTIM‐extracellular‐domain IgFc fusion protein (solid line). Staining was detected with a goat anti‐human‐IgG‐FITC polyclonal antibody. The cells were gated on FSC and SSC plot to exclude dead cells and cell debris. Results are representative from one of four experiments. (b) and (c) The ligand(s) for chTIM4 are up‐regulated after stimulation, as measured by flow cytometry. Splenocytes were cultured without or with (b) recombinant chicken CD40 ligand (rchCD40L) or (c) concanavalin A (ConA) for 48 hr. The cells were co‐stained with chTIM4‐extracellular‐domain IgFc fusion protein conjugated to Alexa‐647, human IgFc protein as a negative control, and either (b) anti‐Bu‐1‐FITC or (c) anti‐CD3‐FITC mAbs. Results show the whole population of cells without any gating and are representative from one of three experiments.

Splenocytes were then stained with either extracellular‐domain‐, IgV‐domain‐ or mucin‐domain‐IgFc fusion proteins, with human IgFc protein as a negative control (Fig. 7a). For chTIM1, around 7% of splenocytes stained with the extracellular‐domain IgFc fusion protein. For chTIM4, splenocytes stained strongly with the extracellular‐domain‐IgFc fusion protein (46·1% positive), weakly with the IgV‐domain‐IgFc fusion protein (4·7% positive) and not at all with the mucin‐domain‐IgFc fusion protein. Following stimulation with rchCD40L, detection of the ligand(s) of chTIM4 on Bu‐1^+^ splenocytes (B cells) was increased (Fig. 7b). Similarly, stimulation of CD3^+^ splenocytes (T cells) with ConA increased binding activity (Fig. 7c).

**Figure 8:**
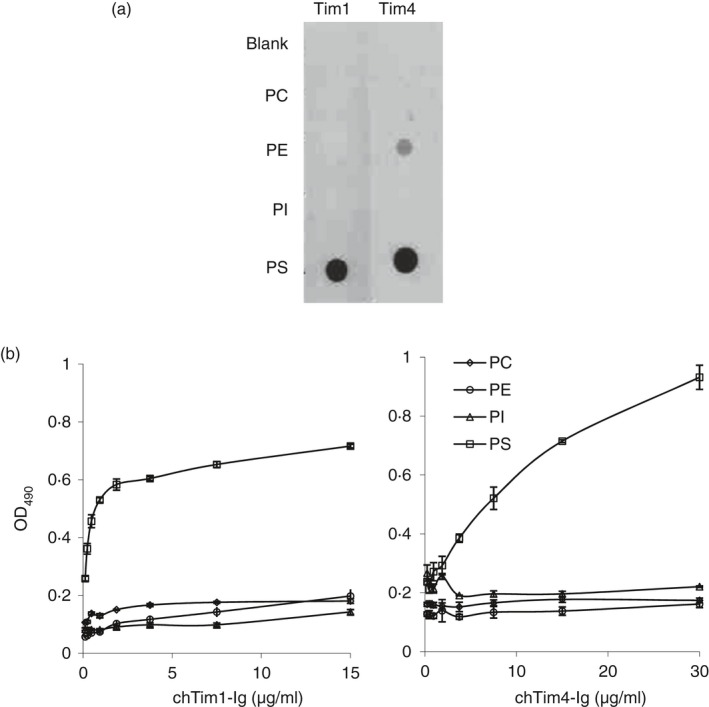
Interaction of chicken T‐cell immunoglobulin and mucin 1 (chTIM1) and chTIM4 with phosphatidyserine (PS) by (a) dot blot, in which phospholipids, phosphatidylcholine (PC), phosphatidylethanolamine (PE), phosphatidylinositol (PI), PS and PBS as control, were dotted onto nitrocellulose membrane and probed by chTIM1‐ or chTIM4‐Ig fusion protein in COS‐7 supernatants, followed by anti‐immunoglobulin antibody, this experiment was repeated twice with different batch of COS‐7 supernatants; and (b) solid‐phase ELISA, in which phospholipids PC, PE, PI and PS, were added to ELISA plates and air dried, and probed by purified chTIM1‐ or chTIM4‐Ig fusion protein at different dilution in triplicate wells, followed by anti‐immunoglobulin antibody. Results are representative from one of three experiments.

**Figure 9:**
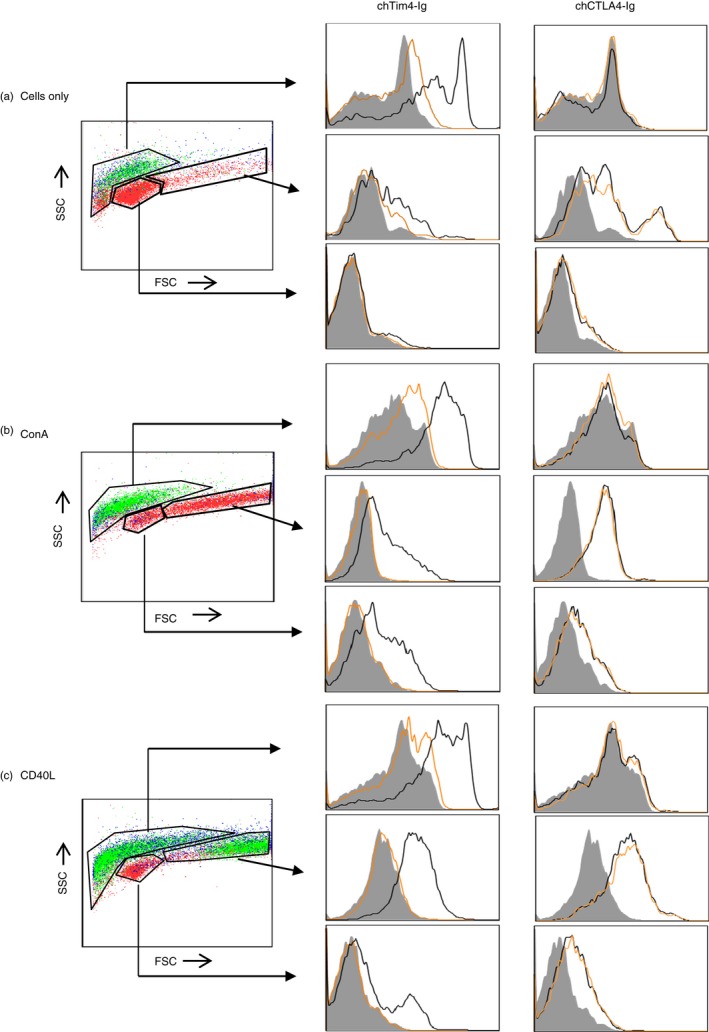
Chicken T‐cell immunoglobulin and mucin 4 (chTIM4) recognizes phosphatidylserine (PS) exposed on apoptotic splenocytes. Splenocytes were cultured alone (a) or with concanavalin A (ConA) (b) or recombinant chicken CD40 ligand (rchCD40L) (c) for 48 hr. In the left‐most panels, red represents unstained cells, blue shows annexin V‐FITC staining and green shows chTIM4‐extracellular‐domain IgFc fusion protein staining. The cells were then stained (left column of histograms) with chTIM4‐extracellular‐domain IgFc fusion protein (black lines), the same protein pre‐incubated with PS for 1 hr (orange lines) or IgFc fusion protein alone as a negative control (filled histogram). Cells were also stained similarly, but with chCTLA4‐extracellular‐domain IgFc fusion protein, as another negative control (right column of histograms). Binding was detected with a rabbit‐anti‐human IgG‐FITC polyclonal antibody. Different cell populations were gated by side and forward scatters. For each treatment, the top panel of histograms show dead cells, the middle early apoptotic cells and the bottom panels live cells.

In mammals, as well as interacting with each other to potentiate Th2 responses through APC–Th2 CD4 T‐cell interactions, TIM1 and TIM4 bind PS and mediate the entry of enveloped viruses such as Ebola^33^, ^34^ and dengue,^35^ and in PS‐mediated phagocytic engulfment and removal of apoptotic cells.^35^ The chTIM4 bound strongly to PS, weakly to PE, and did not bind to PC, PI or the negative control (PBS) in a dot blot assay (Fig. 8a). The chTIM4‐PS interaction was also confirmed in a solid‐phase ELISA in a dose‐dependent manner, but the interactions with PE were not detectable, possibly because of the lower affinity (Fig. 8b). The chTIM1 strongly bound to PS but not to other phospholipids in both assays (Fig. 8a,b). We then tested the ability of chTIM4 to recognize dying cells. Splenocytes were cultured alone (Fig. 9a) or with ConA (Fig. 9b) or rchCD40L (Fig. 9c) for 48 hr. In all three cell populations, live cells were unstained, but dying (early apoptotic) and dead cells stained progressively more strongly with chTIM4. This staining was largely ablated following pre‐incubation of chTIM4 with PS.

### ChTIM4 is co‐stimulatory for chicken splenocytes

**Figure 10:**
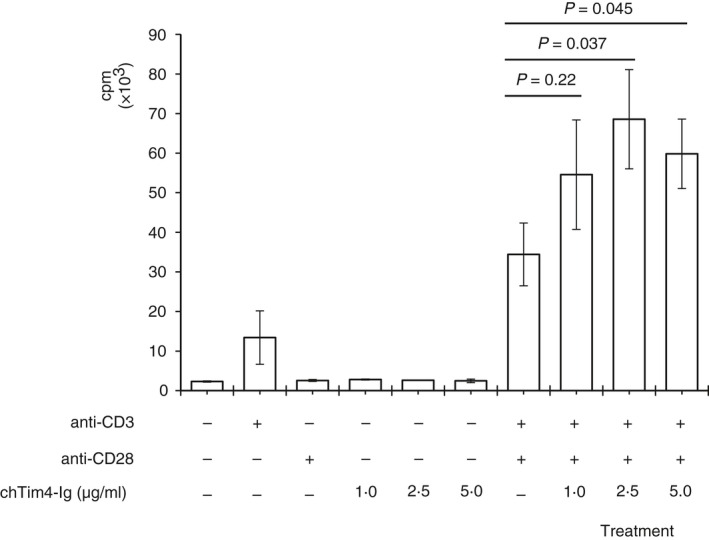
Chicken T‐cell immunoglobulin and mucin 4 (chTIM4) is co‐stimulatory for splenocytes. Splenocytes from SPF line 7_2_ chickens were cultured in complete RPMI‐1640 medium without or with the addition of chTIM4‐extracellular‐domain IgFc fusion protein (chTIM4‐Ig) for 48 hr in wells pre‐coated without or with anti‐chicken CD3 or/and CD28 antibodies. Error bars represent mean ± SEM of triplicate wells for each treatment. The pooled results from four experiments are shown and the *P* value was calculated using Student's *t*‐test when compared with the cells stimulated only by CD3 and CD28 antibodies.

Based upon the known co‐stimulatory activity of TIM protein in mammals, we tested the ability of chTIM4‐extracellular‐domain‐IgFc fusion protein to increase the response of chicken cells to sub‐optimal concentrations of anti‐chicken CD3 and CD28 mAbs for 48 hr. The chTIM4 produced a clear dose‐dependent co‐stimulation of thymidine incorporation in the chicken splenocytes (Fig. 10).

## Discussion

We identified the chicken TIM molecules based on well‐studied mammalian TIMs. The murine TIM family was originally located within an airway hyper‐reactivity regulatory locus on chromosome 11,^31^ syntenic to human chromosomal region 5q23‐35, which contains the human TIM family genes. Both regions are linked to atopy and asthma.^36^, ^37^, ^38^ The human 5q region contains a large number of cytokine genes, including IL‐9, IL‐12 p40 and the Th2 cytokine cluster, which contains the genes that encode IL‐4, IL‐5 and IL‐13, as well as GM‐CSF and IL‐3.^36^, ^39^ Chromosome 13, where the TIM genes are located, appears to be the syntenic chicken chromosome, and encodes IL‐9, IL‐12 p40, the Th2 cytokine cluster (IL‐4, IL‐5 and IL‐13), GM‐CSF and IL‐3.^18^, ^40^, ^41^, ^42^ Like mammals, TIM families in avian species seem to be diverse, with TIM1, TIM3 and TIM4 in a wild bird (zebrafinch) but only TIM1 and TIM4 in the domestic birds (turkey and chicken). Chromosome 13 regions have been linked to genetic control of innate and acquired immunity in layer birds.^43^ It would be of some interest to determine if there is within‐species variation in TIM1 and TIM4 expression levels that could contribute to immune phenotypes.

Murine and human TIM1 and TIM4 are immune‐related molecules, most highly expressed at the mRNA level in immune cells and lymphoid organs^4^, ^44^, ^45^, ^46^ (www.biogps.org), but with no detectable expression in non‐lymphoid tissues, except for high TIM1 expression in the kidney.^2^ By contrast, the chicken TIM1 and TIM4 mRNAs were highly expressed in the immune and non‐immune tissues tested. The difference may be partly explained by the fact that the chicken immune system does not have lymph nodes. Instead, the chicken develops diffuse lymphoid aggregates in organs.^47^ Within leucocyte populations, the expression of chTIM1 and chTIM4 resembles mammalian patterns, including significant T‐cell expression. Notably, chTCR1^+^ cells, which are *γδ* T cells, also highly expressed chTIMs. The biological function of these cells is unclear, but they are clearly capable of cytotoxic activity *in vitro*.^48^

The data from mouse suggest that TIM1–TIM4 interaction promotes T‐cell activation,^10^ and that both can bind PS and contribute to clearance of apoptotic cells.^9^ We demonstrated that chTIM4 fusion protein bound to dying cells, and that binding was also increased upon activation of target cells (Fig. 9). Activation stimuli can also induce PS exposure on different immune cells and strongly depends on the amount of presented antigens.^49^, ^50^ The binding was abolished by pre‐incubation of the protein with PS. Crystallographic analysis of mammalian TIM4 revealed that PS penetrated into a metal‐ion‐dependent ligand binding site in the TIM4‐IgV domain and co‐ordinated with the metal ion.^30^ The PS binding domain is conserved in the chicken protein. TIM4 bound to PS mediated uptake of apoptotic cells and mutations in the metal‐ion‐dependent ligand binding site eliminated PS binding and phagocytosis.^9^ However, TIM4 has no signal transduction motif in its cytoplasm domain. Therefore, it is not able to initiate particle uptake, but rather tethers the apoptotic debris allowing cooperation with other receptors to mediate internalization.^51^

Administration of murine TIM4‐Ig fusion protein *in vivo* with antigen produces increased basal T‐cell proliferation and enhanced production of IL‐2 and interferon‐*γ*.^10^ *In vitro* studies have produced contrasting conclusions. In the presence of anti‐CD3 and anti‐CD28 antibodies for pre‐activation of naive T cells, TIM4‐Ig induced dramatically higher levels of proliferation and cytokine production than those stimulated with a control protein, as well as an increased phosphorylation of TIM1. TIM4 was therefore defined as a co‐stimulatory molecule that promotes T‐cell expansion and survival by cross‐linking TIM1 on activated T cells.^10^, ^11^ By contrast, murine TIM4‐Ig inhibited the proliferation of naive T cells, on which there is very low or no TIM1 expression, possibly by binding to an unknown ligand.^12^ The chicken TIM4‐Ig fusion protein induced splenocyte proliferation in the presence of anti‐chicken CD3 and CD28 antibodies. Although it is not determined which T‐cell subset(s) proliferated, this result suggests that the chicken TIM4 molecule has a function in the regulation of T‐cell immune responses.

In summary, the chicken has fewer TIM family members than mammals, but the functions of the chicken members appear conserved. Uniquely, alternative splicing of chTIM4 generates multiple transcripts which show some preferential expression in different strains of chicken. The chicken lacks many important components of mammalian Th2‐associated responses and Th2‐associated allergy is rarely seen in chickens. It remains to be determined whether chTIM1 and chTIM4 contribute to the regulation of avian cell‐mediated immunity *in vivo*.

## Disclosures

No conflict of interest reported.

## Supporting information


**Figure S1.** Expression of chicken T‐cell immunoglobulin and mucin–immunoglobulin (chTIM Ig) fusion proteins.
