# Supplementary material for: Functional annotation of the T‐cell immunoglobulin mucin family in birds
Source: Immunology. 2016 Jun 20;148(3):287–303. doi: 10.1111/imm.12607 (PMC4913284; doi:10.1111/imm.12607)
Supplement: Supplementary file 1 — Figure S1. Expression of chicken T‐cell immunoglobulin and mucin–immunoglobulin (chTIM Ig) fusion proteins. [file IMM-148-287-s001.docx]

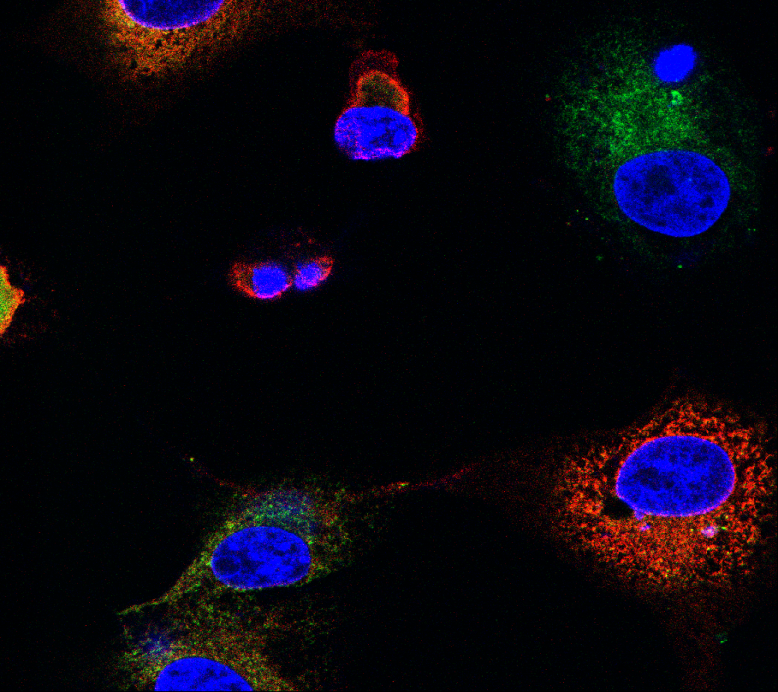

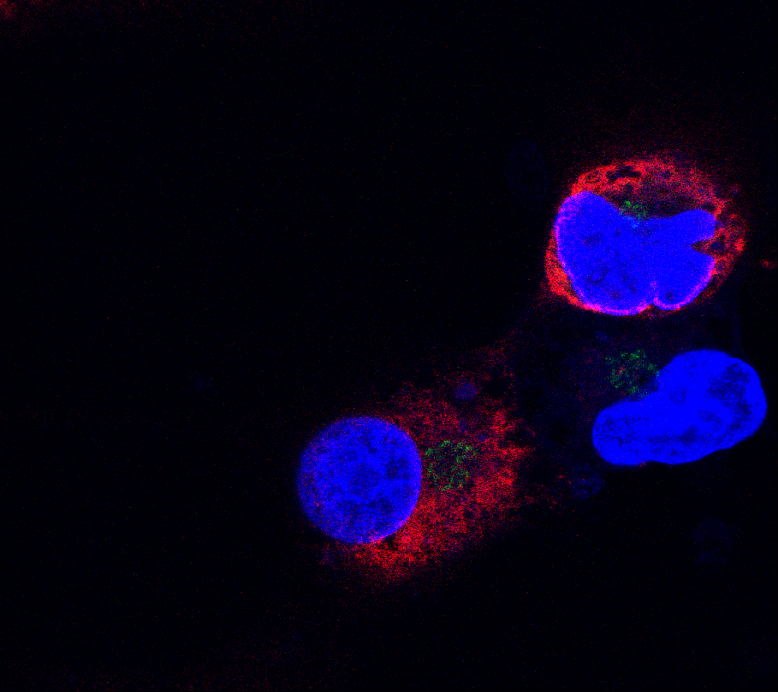


A

i

ii

OD_490_

Supernatant dilutions


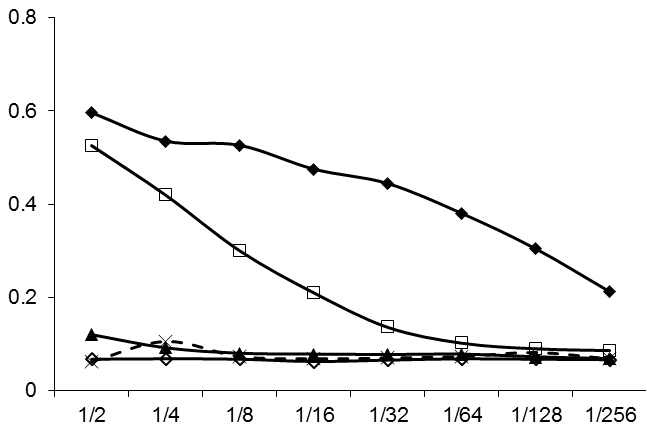


chTIM1

chTIM4

chTIM4L_1_

chTIM4L_1_-2IgV

Negative control

B

**Supplementary Figure 1.** Expression of chTIM Ig-fusion proteins. **A)** Soluble fusion proteins in COS-7 cell supernatants, as measured by capture ELISA. **B)** Confocal microscopy of co-localisation of chTIM4L_1_-extracellular-Ig fusion protein in COS-7 cells. Transfected cells were co-stained with a biotinylated goat anti-human IgG Ab, followed by Alexa-fluor 568-conjugated streptavidin in red, and either (i) mouse anti-PDI (for ER apparatus) or (ii) mouse anti-GM130 (for Golgi apparatus), followed by detection using Alexa-fluor 488-conjugated goat anti-mouse Ab in green. Nuclei were stained with DAPI in blue. Therefore, negatively-transfected cells are in green and blue; positively-transfected cells are in orange and blue (i) or in red, green and blue (ii).
